# Knowledge, attitudes and perceptions of Latin American healthcare workers relating to antibiotic stewardship and antibiotic use: a cross-sectional multi-country study

**DOI:** 10.1186/s13756-024-01400-w

**Published:** 2024-04-26

**Authors:** Valeria Fabre, Sara E. Cosgrove, Fernanda C. Lessa, Twisha S. Patel, Guadalupe Reyes-Morales, Washington R. Aleman, Andrea Alvarado Alvarez, Bowen Aquiles, Ana B. Arauz, Facundo Arguello, Maria Fernanda Barberis, Laura Barcan, Maria P. Bernachea, Marisa L. Bernan, Carlos Buitrago, Maria Del Carmen Bangher, Ximena Castañeda, Angel M. Colque, Alfredo Canton, Rosa Contreras, Silvia Correa, Gustavo Costilla Campero, Lidia Espinola, Clara Esquivel, Cecilia Ezcurra, Leandro A. Falleroni, Johana Fernandez, Sandra Ferrari, Natalia Frassone, Carlos Garcia Cruz, Maria Isabel Garzón, Carlos H. Gomez Quintero, José A. Gonzalez, Lucrecia Guaymas, Fausto Guerrero-Toapanta, Sandra Lambert, Diego Laplume, Paola R. Lazarte, César G. Lemir, Angelica Lopez, Itzel L. Lopez, Herberth Maldonado, Guadalupe Martinez, Diego M. Maurizi, Mario Melgar, Florencia Mesplet, Carlos Morales Pertuz, Cristina Moreno, Gabriela L. Moya, Yanina Nuccetelli, Glendys Núñez, Carolina Osuna, Belén Palacio, Florencia Pellice, Carla Raffo, Fanny Reino Choto, Gerardo Ricoy, Viviana Rodriguez, Federico Romero, Juan J. Romero, Maria Eugenia Russo, Graciela Sadino, Nancy Sandoval, Mirta G. Silva, Alejandra M. Urueña, Ligia Vence Reyes, Hugo Videla, Marisol Valle, Silvia Vera Amate Perez, Hernan Vergara-Samur, Silvina Villamandos, Olmedo Villarreal, Alejandra Viteri, Eduardo Warley, Rodolfo E. Quiros

**Affiliations:** 1 Department of Medicine, Division of Infectious Diseases, Johns Hopkins University School of Medicine, 600 North Wolfe Street, Halstead 824, Baltimore, MD 21287 USA; 2 International Infection Control Program, Division of Healthcare Quality Promotion, National Center for Emerging and Zoonotic Infectious Diseases, Centers for Disease Control and Prevention, Atlanta, GA USA; 3 Hospital Alcivar, Guayaquil, Ecuador; 4 Unidad de Cirugía Cardiovascular de Guatemala, Guatemala City, Guatemala; 5 Hospital Sociedad de Lucha Contra El Cáncer, Guayaquil, Ecuador; 6 Departamento de Medicina, Universidad de Panamá, Panama, Panama; 7 Hospital Santo Tomas, Panama, Panama; 8 Hospital Italiano de Buenos Aires, Buenos Aires, Argentina; 9 Hospital Nacional Profesor Alejandro Posadas, El Palomar, Argentina; 10 Clínica Conciencia, Neuquén, Argentina; 11 Hospital Interzonal General de Agudos San Roque, La Plata, Argentina; 12 Pacifica Salud, Hospital Punta Pacifica, Panamá, Panama; 13 Instituto de Cardiología de Corrientes “Juana Francisca Cabral”, Corrientes, Argentina; 14 Clínica De La Mujer, Bogotá, Colombia; 15 Hospital Medico Policial Churruca Visca, Buenos Aires, Argentina; 16 Hospital Dr. Marcial V. Quiroga, San Juan, Argentina; 17 Hospital Municipal de Trauma Dr. Federico Abete, Malvinas Argentinas, Argentina; 18 Hospital Angel C. Padilla, Tucumán, Argentina; 19 Hospital El Cruce, Buenos Aires, Argentina; 20 Hospital San Benito, Peten, Guatemala; 21 Hospital Alemán, Buenos Aires, Argentina; 22 Hospital Provincial de Rosario, Rosario, Argentina; 23 Hospital Dr. Guillermo Rawson, San Juan, Argentina; 24 Clínica Universitaria Privada Reina Fabiola, Córdoba, Argentina; 25 Hospital Privado Universitario de Córdoba, Córdoba, Argentina; 26 Hospital Militar Central, Bogotá, Colombia; 27 Hospital Irma de Lourdes Tzanetatos, Panama, Panama; 28 Clinica Privada Provincial, Buenos Aires, Argentina; 29 Hospital Carlos Andrade Marín, Quito, Ecuador; 30 Maternidad Nuestra, Tucumán, Argentina; 31 Hospital San Bernardo, Salta, Argentina; 32 Clinica Hospital San Fernando, Panama, Panama; 33 Hospital Municipal de Agudos Dr. Leonidas Lucero, Bahía Blanca, Argentina; 34 Hospital Roosevelt, Guatemala, Guatemala; 35 Hospital Cesar Milstein, Buenos Aires, Argentina; 36 Hospital Del Tunal, Bogotá, Colombia; 37 Hospital Metropolitano, Quito, Ecuador; 38 Instituto de Diagnostico, La Plata, Argentina; 39 Hospital Zonal General de Agudos Dr. Alberto Eurnekian, Buenos Aires, Argentina; 40 Sanatorio Allende Nueva Córdoba, Córdoba, Argentina; 41 Hospital Vozandes, Quito, Ecuador; 42 The Panama Clinic, Panama, Panama; 43 Sanatorio Las Lomas, Buenos Aires, Argentina

**Keywords:** Antibiotic, Stewardship, Latin America

## Abstract

**Background:**

The burden of antimicrobial resistance (AMR) in Latin America is high. Little is known about healthcare workers’ (HCWs) knowledge, attitudes, and perceptions of antimicrobial stewardship (AS), AMR, and antibiotic use (AU) in the region.

**Methods:**

HCWs from 42 hospitals from 5 Latin American countries were invited to take an electronic, voluntary, anonymous survey regarding knowledge, attitudes, and perceptions of AS, AMR, and AU between March–April 2023.

**Findings:**

Overall, 996 HCWs completed the survey (52% physicians, 32% nurses, 11% pharmacists, 3% microbiologists, and 2% “other”). More than 90% of respondents indicated optimizing AU was a priority at their healthcare facility (HCF), 69% stated the importance of AS was communicated at their HCF, and 23% were unfamiliar with the term “antibiotic stewardship”. Most (> 95%) respondents acknowledged that appropriate AU can reduce AMR; however, few thought AU (< 30%) or AMR (< 50%) were a problem in their HCF. Lack of access to antibiogram and to locally endorsed guidelines was reported by 51% and 34% of HCWs, respectively. Among prescribers, 53% did not consider non-physicians’ opinions to make antibiotic-related decisions, 22% reported not receiving education on how to select antibiotics based on culture results and 60% stated patients and families influence their antibiotic decisions.

**Conclusions:**

Although HCWs perceived improving AU as a priority, they did not perceive AU or AMR as a problem in their HCF. AS opportunities include improved access to guidelines, access to AMR/AU data, teamwork, and education on AS for HCWs and patients and families.

**Supplementary Information:**

The online version contains supplementary material available at 10.1186/s13756-024-01400-w.

## Introduction

Optimizing antimicrobial use (AU) is a key strategy to prevent and reduce antimicrobial resistance (AMR) [1]. Previous studies have identified several determinants of hospital AU including cultural (e.g., socioeconomic), contextual (e.g., local antibiotic restriction policies), and behavioral (e.g., attitudes towards antibiotics upon certain clinical scenarios or patient population) aspects [2].

A recent study conducted by the same research group explored barriers to implementation of effective antimicrobial stewardship programs (ASPs) in Latin American hospitals through self-assessment questionnaires and interviews with antibiotic stewardship (AS) stakeholders and found several challenges including limited availability of clinical pharmacists, lack of protected time for physicians, pharmacists, or microbiologists to perform AS activities as well as limited information and technology resources to track and analyze antibiotic or AMR data [3]. However, HCWs attitudes and perceptions concerning AS and AU in Latin America remain largely unknown. To address this gap, we conducted a multi-center cross-sectional survey among HCWs including both prescribers and non-prescribers from acute care hospitals from five Latin American countries.

## Methods

### Study setting and population

HCWs from 42 acute care hospitals in Guatemala, Panama, Ecuador, Colombia, and Argentina (25 non-profit and 17 for-profit) were invited to complete an anonymous voluntary electronic survey. Recruitment for survey participation was performed by the local AS teams, who were also responsible for distributing the electronic survey. The target audience included physicians, nurses, pharmacists, and microbiologists, although other healthcare roles such as physical or respiratory therapists could be included. Local AS teams were recommended to aim for a minimum of 30 responses. This survey was conducted as part of a larger project to evaluate current state of ASPs in Latin America. Country selection was discussed with CDC officers and national public health authorities to ensure study activities were not conflicting with other ongoing activities related to AS in the country. Hospitals were recruited through a regional research network (PROAnet) [3]. Requirement for study participation included having an individual(s) responsible for leading and/or performing AS activities in the hospital. A list of participating hospitals is included in Supplementary material.

### Survey development

The survey was developed by a multidisciplinary team including physicians, pharmacists, microbiologists, implementation scientists, and public health officials, all with experience in AS. Investigators developed an initial list of qualitative assessment items based on experience and literature review. These questions were further reviewed applying the Delphi method. The survey was pilot tested by nine Latin American HCWs to assess the time necessary to fill out the entire questionnaire, ensure questions were relevant, and test the clarity of the questions. Four questions were modified as a result of their feedback for better clarity. The questionnaire included 20 questions relating to knowledge, attitudes, and perceptions about AS, AMR and AU. Additionally, prescribers, were given 21 additional questions about antibiotic decision-making and factors influencing this process. The questionnaire was built using the Qualtrics survey system (Qualtrics, Provo, UT, USA). The survey was translated to Spanish by a certified translator and verified by 2 bilingual native Spanish speaking investigators (VF, REQ) to ensure the translation maintained the original meaning (see survey in both languages in Supplementary material).

Responses on a 5-point Likert scale were collapsed into 2 categories, (e.g., strongly agree/agree versus neutral/disagree/strongly disagree) [4]. Data were analyzed using chi-square test in STATA version 16.0 software (StataCorp, College Station, TX). A 2-sided *P* value < 0.05 was considered statistically significant. Results were analyzed overall and by healthcare worker role.

## Results

### Participant characteristics

Nine hundred and ninety-six HCWs completed the survey, including 519 physicians (52%), 324 nurses (32%), 109 pharmacists (11%), 28 microbiologists (3%), and 16 (2%) individuals in other healthcare roles. Four-hundred and eighty-nine physicians (49%) were self-identified as prescribers. Overall, the median years of experience working at the HCF was 8 (interquartile range [IQR] 3, 15), and 202 (21%) of respondents were in training.

### Knowledge, attitudes, and perceptions about antibiotic stewardship and antibiotic decision-making

Across roles, most (> 90%) participants indicated that optimizing antibiotic use was a priority for their healthcare facilities (HCFs). However, 69% stated that the importance of AS was communicated at their HCFs, and 77% were familiar with the term “antibiotic stewardship”. Using antibiotics appropriately was acknowledged as a strategy to reduce AMR by 95% of respondents. Less than 30% of respondents thought antibiotics were overused and less than 50% thought AMR was a problem at their HCF. These perceptions varied by role.

Overall, 75% of respondents stated they value the input by the AS team, including 78% of prescribers (382/489). While 75% of physicians reported feeling comfortable recommending changes to antibiotics to other HCWs, significantly fewer nurses (39%) and pharmacists (52%) agreed with this statement (*P* = 0.000). There was overall high agreement (~ 80%) that antibiotic decisions are conducted by multidisciplinary teamwork; however, there were significant differences in responses by role (fewer pharmacists and nurses compared to physicians were in agreement with the statement [69% and 73% respectively vs. 83%, *P* = 0.001]). Respondents with more years of work experience were more likely to indicate there was multidisciplinary teamwork related to antibiotic decision-making than those with fewer years of work experience (81% vs. 72%, *P* = 0.002). Furthermore, only 47% of prescribers indicated they considered the opinions of non-physicians including nurses or pharmacists when making antibiotic decisions (regardless of years of experience).

Access to locally endorsed treatment guidelines was reported by 74% of physicians, 53% of nurses and 56% of pharmacists.

Responses to all 20 questions (overall, and by role) are presented in Table 1.

**Table 1:** Agreement with statements relating to knowledge, perceptions of and attitudes towards antibiotic use, antimicrobial resistance, and antibiotic stewardship among 996 healthcare workers, overall and by role. Agreement includes “strongly agree” and “agree” responses

Question	Overall *N* = 996 (%)	Physician * N* = 519 (%)	Nurse *N* = 324 (%)	Pharmacist *N* = 109 (%)	Other *N* = 44 (%)	*P* value*	*P* value**
Optimizing AU is a priority at my HCF	934 (93.8)	487 (93.8)	299 (92.3)	105 (96.3)	43 (97.7)	0.304	0.936
AU is discussed at facility-wide multidisciplinary meetings	820 (82.3)	449 (86.5)	253 (78.1)	86 (78.9)	32 (72.7)	**0.003**	**0.000**
I am familiar with the term “antibiotic stewardship”	772 (77.5)	415 (79.9)	226 (69.7)	96 (88.1)	35 (79.5)	**0.000**	0.05
The importance of AS is communicated at my HCF	690 (69.3)	397 (76.5)	208 (64.2)	65 (59.6)	20 (45.4)	**0.000**	**0.000**
I trust the microbiology test results that I receive at my HCF	905 (90.9)	488 (94.0)	280 (86.4)	96 (88.1)	41 (93.2)	**0.002**	**0.000**
My HCF promptly alerts prescribers about relevant positive culture results to modify antibiotic therapy	839 (84.2)	451 (86.7)	268 (82.7)	81 (74.3)	39 (88.6)	**0.007**	**0.01**
I am able to access my HCF's updated antibiogram	504 (50.6)	284 (54.7)	152 (46.9)	43 (39.4)	25 (56.8)	**0.01**	**0.07**
Use of broad-spectrum antibiotics when equally effective narrower spectrum antibiotics are available increases AMR	883 (88.6)	493 (94.9)	257 (79.3)	96 (88.1)	37 (84.1)	** < 0.001**	**0.000**
Inappropriate antibiotic use can harm patients	989 (99.3)	518 (99.8)	319 (98.5)	109 (100)	43 (97.7)	0.05	**0.04**
The incidence of antibiotic-resistant organisms can be reduced by optimizing antibiotic prescribing patterns and infection prevention and control practices	978 (98.2)	526 (99.4)	312 (96.3)	107 (98.2)	43 (97.7)	**0.012**	**0.02**
Appropriate use of antibiotics may reduce antibiotic resistance	977 (98.1)	515 (99.2)	311 (95.9)	109 (100)	42 (95.4)	**0.002**	**0.006**
Requiring clinicians to obtain approval prior to prescribing certain antibiotics is a way to improve AU	923 (92.7)	477 (91.9)	300 (92.6)	104 (95.4)	42 (95.4)	0.541	0.335
Antibiotics are overused at my HCF	273 (27.4)	190 (36.6)	46 (14.2)	28 (25.7)	9 (20.4)	**0.000**	**0.000**
Antibiotic resistance is a problem at my HCF	465 (46.7)	295 (56.8)	90 (27.8)	61 (66.9)	19 (43.2)	**0.000**	**0.000**
There is multidisciplinary teamwork for antibiotic decision-making activities at my HCF	774 (77.7)	430 (82.8)	236 (72.8)	75 (68.8)	33 (75)	**0.001**	**0.000**
I value recommendations from the AS team at my HCF	751 (75.4)	404 (77.8)	235 (72.5)	81 (74.3)	31 (70.4)	0.289	0.06
I have access to locally endorsed ID treatment guidelines	641 (64.4)	383 (73.8)	172 (53.1)	61 (55.9)	25 (56.8)	**0.000**	**0.000**
I have adequate access to ID expertise at my HCF	906 (90.9)	497 (96.7)	282 (87.0)	89 (81.6)	38 (86.4)	**0.000**	**0.000**
I feel comfortable recommending an intervention to my colleagues on AU	589 (59.1)	390 (75.1)	125 (38.6)	57 (52.3)	17 (38.6)	**0.000**	**0.000**
HCWs educate patients and/or their families on the use of antibiotics at discharge at my HCF	643 (64.5)	319 (61.5)	233 (71.9)	63 (57.8)	28 (63.6)	**0.007**	**0.03**

*AU* antibiotic use, *HCF* healthcare facility, *AMR* antimicrobial resistance, *AS* antibiotic stewardship, *ID* infectious diseases

^*^Refers to overall *P* value of the chi-square test comparing strongly agree/agree vs. neutral/disagree/strongly disagree for the different roles overall

^**^Refers to the *P* value of the chi-square test comparing physician vs. non-physician roles

### Factors influencing antibiotic prescribing

Among 489 prescribers, 60% reported that patients and/or their families may influence their prescribing practices while 36% reported they are pressured by their colleagues to prescribe antibiotics. Additional factors influencing antibiotic decisions reported by prescribers included: scientific literature (89%), ID consultation (85%), and local guidelines (82%). Only 55% of prescribers reported having access to their HCF antibiograms. Prescribers with access to HCF antibiograms were more likely to consider the risk of AMR development when prescribing antibiotics than those who did not (57% vs. 43%,* P* = 0.003).

While most (97.5%) prescribers stated they would modify antibiotics based on culture results, 18% indicated not routinely getting cultures (when indicated) before starting antibiotics.

Most (93%) prescribers perceived feedback as a strategy to improve their prescribing practices, and 74% thought comparing their prescribing rates with others would be helpful to modify their antibiotic behavior. Although 96% of prescribers stated more education could improve their antibiotic prescribing practices, 22% indicated they had not received education on how to make appropriate antibiotic choices regardless of years of experience.

Responses to the additional 21 questions targeting prescribers are presented in Table 2.

**Table 2:** Agreement with statements relating to antibiotic prescribing among 489 prescribers. Agreement includes “Strongly agree” and “agree” responses

Statement	Strongly agree/agree N (%)
More guidance from the AS team could improve AU	459 (93.9)
I have timely access to microbiology test results and diagnostic information to guide my use of antibiotics	443 (90.6)
Receiving more education on appropriate selection of antibiotic agent, duration of therapy, and dose could improve my antibiotic prescribing practices at my HCF	470 (96.1)
I receive education on how to select the most appropriate antibiotic for treatment based on microbiology test results at my HCF	385 (78.7)
The AS team can impact my decisions on antibiotic initiation and continuation at my HCF	457 (93.5)
I am pressured to prescribe antibiotics by patients or their families	295 (60.3)
I am pressured to prescribe antibiotics by my colleagues	174 (35.6)
Scientific literature influences my decisions on antibiotic prescribing at my HCF	438 (89.6)
Pharmaceutical companies influence some of my decisions on antibiotic prescribing at my HCF	67 (13.7)
I use locally endorsed ID treatment guidelines when I am making decisions about antibiotic prescribing at my HCF	400 (81.8)
I prescribe certain empiric antibiotics based on consultation with a clinician with experience practicing ID, ID trained physician, or the AS team at my HCF	414 (84.6)
I routinely obtain cultures before starting antibiotic therapy in patients with suspected infection at my HCF	404 (82.6)
I modify my patient's antibiotic treatment after receiving culture and antibiotic susceptibility results when appropriate	477 (97.5)
I consider adverse events when selecting an antibiotic regimen for patients at my HCF	468 (95.7)
I consider drug interactions when selecting an antibiotic regimen for a defined patient population at my HCF	444 (90.8)
I consider my patient's kidney function when dosing antibiotics at my HCF	475 (97.1)
I consider the risk of development of antibiotic resistance in my patients when I prescribe antibiotics	442 (90.4)
I consider the opinion of non-physician staff (e.g., nursing, pharmacy) in antibiotic decision-making at my HCF	219 (44.8)
Receiving feedback about appropriateness of antibiotics that I prescribe could improve my antibiotic prescribing practices	457 (93.5)
Receiving feedback on how my antibiotic prescribing practices compares to my peers could improve my antibiotic prescribing practices	364 (74.4)
I am aware of changes that are needed to my current antibiotic prescribing practices based on feedback received at my HCF	438 (89.6)

*AS* antibiotic stewardship, *AU* antibiotic use, *HCF* healthcare facility

## Discussion

We conducted a cross-sectional survey among 996 HCWs from 5 countries in Latin America to explore their knowledge, perceptions and attitudes towards AU, AMR, and AS. We found their perceptions to be similar to those reported in other settings and countries where HCWs recognize AMR or AU as an important topic, but don’t necessarily perceive it as a problem in their own hospital or practice [5, 6].

In our survey, most participants acknowledged optimizing AU was a priority at their HCF; yet, almost a quarter of respondents were not familiar with the term “antibiotic stewardship”, and a third reported the importance of AS was not communicated at their HCF. Participants reported limited access to the HCF antibiogram, and limited education on AS which may account for lower awareness of AS or AMR as a local problem. A recent study conducted by the same research group on the current state of ASP implementation in Latin American hospitals identified limited access to robust information and technology resources as well as insufficient personnel in both the clinical microbiology laboratory and the AS teams as barriers to collecting and reporting aggregate data such as the HCF antibiogram or antibiotic use data which might explain survey findings [3].

There are several strategies ASPs can implement to modify AU in the hospital setting such as post-prescription review with feedback, restriction of broad-spectrum antimicrobials, automatic stops, time-outs, and treatment guidelines, all of which require multidisciplinary teamwork including integration of pharmacists and nurses who can provide unique and complementary information to physicians for management decisions [7–11]. In our survey, 40% of participants did not feel comfortable making suggestions regarding antibiotics to a colleague, and > 50% of prescribers did not value the opinions of non-physicians. These findings coupled with pharmacists’ limited access to healthcare facility treatment guidelines and limited availability of pharmacists trained in clinical pharmacy or infectious diseases underscore significant barriers to pharmacists taking a more active role in AS in the region. Nurses were less likely to perceive AMR or AU as a problem compared to physicians and pharmacists which may suggest limited integration of nurses in AS activities. According to a recent self-assessment of AS activities, implementation of strategies to promote multidisciplinary teamwork were uncommon in Latin American hospitals [3]. Additional barriers to interdisciplinary work in AS identified in studies conducted in the region included hierarchical relationships [3, 12, 13].

Treatment guidelines remain a core strategy to optimize antibiotic use. Limited access to locally adapted treatment guidelines was reported by 30–40% of HCWs in this survey; however, most people with access to guidelines reported using them. Over half of prescribers taking the survey reported pressure to prescribe antibiotics by patients and/or patients’ families; however, it is unclear whether this remains a major challenge in the inpatient setting. While the findings highlight the need to strengthen and expand education on optimal antibiotic use to the community there is also a need to perform more research in this area in the inpatient setting [6, 14].

Limitations of our study include the lack of a response rate due to how the survey was disseminated (local AS teams would share the electronic survey link to key stakeholders in the hospital such as directors, managers, etc. for further survey distribution). Study findings may not be widely generalizable to the region as we used a convenience sample of hospitals recruited through a regional network; hence, participating facilities might be more engaged in AS than hospitals not participating in the network. Similarly, we only included five of the 33 countries in Latin America. Finally, it is possible that respondents gave socially desirable answers and there may be underestimation of potential areas for improvement (e.g., very few prescribers indicated they are influenced by pharmaceutical companies). Strengths of the study include a large number of respondents, inclusion of both prescribers and non-prescribers from a mix of non-profit and for-profit hospitals. Furthermore, many of our observations are consistent with prior studies conducted both in non-limited and limited resource settings validating the results [5, 6].

## Conclusions

In summary, we identified several areas to focus on to strengthen AS in Latin American hospitals including increasing HCWs and community awareness of the importance of AS and optimal AU, improved access to resources such as healthcare facility antibiograms and treatment guidelines. Cultural and behavioral determinants remain critical components of AS improvement efforts.

### Supplementary Information

**Supplementary Material 1.**

## Data Availability

See Supplementary material.
