# Supplementary material for: Knowledge, attitudes and perceptions of Latin American healthcare workers relating to antibiotic stewardship and antibiotic use: a cross-sectional multi-country study
Source: Antimicrob Resist Infect Control. 2024 Apr 26;13:47. doi: 10.1186/s13756-024-01400-w (PMC11045452; doi:10.1186/s13756-024-01400-w)
Supplement: Supplementary file 1 — Supplementary Material 1. [file 13756_2024_1400_MOESM1_ESM.docx]

**Perceptions and Attitudes of Latin American Healthcare Workers Relating To Antibiotic Stewardship and Antibiotic Use: A Cross-Sectional Multi-Country Study. *Fabre et al.***

**SUPPLEMENTARY MATERIAL**

Healthcare worker survey (English)………………………………………………………………… Pages 2-9

Healthcare worker survey (Spanish)………………………………………………………………… Pages 10-17

Participating hospitals………………………………………………………………………………… Pages 17

Survey conducted among healthcare workers to evaluate their knowledge, attitudes and perceptions about antibiotic stewardship, antibiotic use, and antimicrobial resistance (English and Spanish versions).

1. **English version**

Please select your healthcare profession:  physician  pharmacist  nursing staff (e.g., nurse, nurse technician)  other, please specify:

Are you currently in training (e.g., residency, fellowship)?  yes  no

Please enter the number of years of experience that you have in the selected healthcare profession. ________

Please enter the number of years that you have worked at this healthcare facility. _________

Name of hospital:

| **FOR *ALL* HEALTHCARE WORKERS: Please rate your agreement with each of the following statements below.** | | |
| --- | --- | --- |
| 1 | Optimizing antibiotic use is a priority at my healthcare facility. | strongly agree  agree  neutral  disagree  strongly disagree  not applicable |
| 2 | Antibiotic use is discussed at facility-wide multidisciplinary team meetings. | strongly agree  agree  neutral  disagree  strongly disagree  not applicable |
| 3 | I am familiar with the term antibiotic stewardship. | strongly agree  agree  neutral  disagree  strongly disagree  not applicable |
| 4 | The importance of antibiotic stewardship is communicated (e.g., via posters, emails) at my healthcare facility. | strongly agree  agree  neutral  disagree  strongly disagree  not applicable |
| 5 | I trust the microbiology test results that I receive at my healthcare facility. | strongly agree  agree  neutral  disagree  strongly disagree  not applicable |
| 6 | My healthcare facility promptly alerts prescribers about relevant positive culture results (e.g., organism identified in blood culture) to modify antibiotic therapy. | strongly agree  agree  neutral  disagree  strongly disagree  not applicable |
| 7 | I am able to access my healthcare facility's updated antibiogram. | strongly agree  agree  neutral  disagree  strongly disagree  not applicable |
| 8 | Use of broad-spectrum antibiotics when equally effective narrower spectrum antibiotics are available increases antibiotic resistance. | strongly agree  agree  neutral  disagree  strongly disagree  not applicable |
| 9 | Inappropriate antibiotic use can harm patients. | strongly agree  agree  neutral  disagree  strongly disagree  not applicable |
| 10 | The incidence of antibiotic-resistant organisms can be reduced by optimizing antibiotic prescribing patterns and infection prevention and control practices. | strongly agree  agree  neutral  disagree  strongly disagree  not applicable |
| 11 | Appropriate use of antibiotics may reduce antibiotic resistance. | strongly agree  agree  neutral  disagree  strongly disagree  not applicable |
| 12 | Requiring clinicians to obtain approval prior to prescribing certain antibiotics is a way to improve antibiotic use. | strongly agree  agree  neutral  disagree  strongly disagree  not applicable |
| 13 | Antibiotics are overused (e.g., antibiotics are used when not clinically indicated) at my healthcare facility. | strongly agree  agree  neutral  disagree  strongly disagree  not applicable |
| 14 | Antibiotic resistance is a problem at my healthcare facility. | strongly agree  agree  neutral  disagree  strongly disagree  not applicable |
| 15 | There is multidisciplinary teamwork for antibiotic decision-making activities at my healthcare facility. | strongly agree  agree  neutral  disagree  strongly disagree  not applicable |
| 16 | I value recommendations from the antibiotic stewardship team at my healthcare facility. | strongly agree  agree  neutral  disagree  strongly disagree  not applicable |
| 17 | I have access to locally endorsed infectious diseases treatment guidelines used at my healthcare facility. | strongly agree  agree  neutral  disagree  strongly disagree  not applicable |
| 18 | I have adequate access to infectious diseases expertise (e.g., infectious diseases trained physician or clinician with experience practicing infectious diseases) at my healthcare facility. | strongly agree  agree  neutral  disagree  strongly disagree  not applicable |
| 19 | I feel comfortable recommending an intervention to my colleagues on antibiotic use. | strongly agree  agree  neutral  disagree  strongly disagree  not applicable |
| 20 | Healthcare workers (e.g., physicians, pharmacists, or nurses) educate patients and/or their families on the use of antibiotics at discharge at my healthcare facility. | strongly agree  agree  neutral  disagree  strongly disagree  not applicable |

| **FOR *PRESCRIBERS* ONLY: Please rate your agreement with each of the following statements below.** | | |
| --- | --- | --- |
| 21 | Receiving more education on appropriate selection of antibiotic agent, duration of therapy, and dose could improve my antibiotic prescribing practices at my healthcare facility. | strongly agree  agree  neutral  disagree  strongly disagree  not applicable |
| 22 | I receive education on how to select the most appropriate antibiotic for treatment based on microbiology test results at my healthcare facility. | strongly agree  agree  neutral  disagree  strongly disagree  not applicable |
| 23 | The antibiotic stewardship team can impact my decisions on antibiotic initiation and continuation at my healthcare facility. | strongly agree  agree  neutral  disagree  strongly disagree  not applicable |
| 24 | I am pressured to prescribe antibiotics by patients or their families. | strongly agree  agree  neutral  disagree  strongly disagree  not applicable |
| 25 | I am pressured to prescribe antibiotics by my colleagues. | strongly agree  agree  neutral  disagree  strongly disagree  not applicable |
| 26 | Scientific literature (e.g., published research) influences my decisions on antibiotic prescribing at my healthcare facility. | strongly agree  agree  neutral  disagree  strongly disagree  not applicable |
| 27 | Pharmaceutical companies influence some of my decisions on antibiotic prescribing at my healthcare facility. | strongly agree  agree  neutral  disagree  strongly disagree  not applicable |
| 28 | I use locally endorsed infectious diseases treatment guidelines when I am making decisions about antibiotic prescribing at my healthcare facility. | strongly agree  agree  neutral  disagree  strongly disagree  not applicable |
| 29 | I prescribe certain empiric antibiotics based on consultation with a clinician with experience practicing infectious diseases, infectious diseases trained physician, or the antibiotic stewardship team at my healthcare facility. | strongly agree  agree  neutral  disagree  strongly disagree  not applicable |
| 30 | I routinely obtain cultures before starting antibiotic therapy in patients with suspected infection at my healthcare facility. | strongly agree  agree  neutral  disagree  strongly disagree  not applicable |
| 31 | I modify my patient's antibiotic treatment after receiving culture and antibiotic susceptibility results when appropriate. | strongly agree  agree  neutral  disagree  strongly disagree  not applicable |
| 32 | I consider adverse events when selecting an antibiotic regimen for patients at my healthcare facility. | strongly agree  agree  neutral  disagree  strongly disagree  not applicable |
| 33 | I consider drug interactions when selecting an antibiotic regimen for a defined patient population at my healthcare facility. | strongly agree  agree  neutral  disagree  strongly disagree  not applicable |
| 34 | I consider my patient's kidney function when dosing antibiotics at my healthcare facility. | strongly agree  agree  neutral  disagree  strongly disagree  not applicable |
| 35 | I consider the risk of development of antibiotic resistance in my patients when I prescribe antibiotics. | strongly agree  agree  neutral  disagree  strongly disagree  not applicable |
| 36 | I consider the opinion of non-physician staff (e.g., nursing, pharmacy) in antibiotic decision-making at my healthcare facility. | strongly agree  agree  neutral  disagree  strongly disagree  not applicable |
| 37 | Receiving feedback about appropriateness of antibiotics that I prescribe could improve my antibiotic prescribing practices. | strongly agree  agree  neutral  disagree  strongly disagree  not applicable |
| 38 | Receiving feedback on how my antibiotic prescribing practices compares to my peers could improve my antibiotic prescribing practices. | strongly agree  agree  neutral  disagree  strongly disagree  not applicable |
| 39 | I am aware of changes that are needed to my current antibiotic prescribing practices based on feedback received at my healthcare facility. | strongly agree  agree  neutral  disagree  strongly disagree  not applicable |

1. **Spanish version (Version Español)**

Seleccione su rol: médico ☐ farmacéutico ☐ personal de enfermería (por ejemplo, enfermera, técnico de enfermería) ☐ otro, especifique: ☐

¿Está actualmente en formación (por ejemplo, residencia, beca)? ☐ Sí No ☐

Introduzca el número de años de experiencia que tiene en la profesión sanitaria seleccionada. ________

Ingrese el número de años que ha trabajado en este centro de atención médica. _________

Nombre del hospital:

| **Por favor califique su acuerdo con cada una de las siguientes declaraciones a continuación.** | | |
| --- | --- | --- |
| 1 | Mejorar el uso de antibióticos es una prioridad en mi centro de salud. | totalmente de acuerdo  de acuerdo  neutral  desacuerdo  totalmente en desacuerdo  no aplica |
| 2 | Temas relacionados con el uso de antibióticos (ej. consumo, indicaciones, etc.) se discuten en reuniones en mi establecimiento de salud con el motivo de mejorar el uso. | totalmente de acuerdo  de acuerdo  neutral  desacuerdo  totalmente en desacuerdo  no aplica |
| 3 | Estoy familiarizado con el término optimización del uso de antibióticos o programas para la optimización del uso de antibióticos (PROA). | totalmente de acuerdo  de acuerdo  neutral  desacuerdo  totalmente en desacuerdo  no aplica |
| 4 | La importancia del uso racional y adecuado de antibióticos se comunica en mi establecimiento de salud (ej. a través de carteles, correos electrónicos, charlas, etc.) | totalmente de acuerdo  de acuerdo  neutral  desacuerdo  totalmente en desacuerdo  no aplica |
| 5 | Confío en los resultados de las pruebas de microbiología que recibo en mi centro de salud. | totalmente de acuerdo  de acuerdo  neutral  desacuerdo  totalmente en desacuerdo  no aplica |
| 6 | En mi establecimiento de salud, se comunica rápidamente los resultados positivos de ciertos cultivos (ej. de los hemocultivos) para modificar el esquema antibiótico. | totalmente de acuerdo  de acuerdo  neutral  desacuerdo  totalmente en desacuerdo  no aplica |
| 7 | Tengo acceso a los reportes anuales agregados de sensibilidad a los antibióticos de mi establecimiento de salud. | totalmente de acuerdo  de acuerdo  neutral  desacuerdo  totalmente en desacuerdo  no aplica |
| 8 | Prescribir un antibiótico de amplio espectro cuando existe un antibiótico de espectro reducido contribuye al desarrollo de resistencia a los antibióticos. | totalmente de acuerdo  de acuerdo  neutral  desacuerdo  totalmente en desacuerdo  no aplicable |
| 9 | El uso inadecuado de antibióticos puede causar daño a los pacientes. | totalmente de acuerdo  de acuerdo  neutral  desacuerdo  totalmente en desacuerdo  no aplica |
| 10 | La incidencia de organismos multiresistentes puede reducirse mejorando el uso de antibióticos y las prácticas de prevención y control de infecciones. | totalmente de acuerdo  de acuerdo  neutral  desacuerdo  totalmente en desacuerdo  no aplica |
| 11 | Un mejor uso de antibióticos puede reducir la resistencia a los antibióticos. | totalmente de acuerdo  de acuerdo  neutral  desacuerdo  totalmente en desacuerdo  no aplica |
| 12 | La restricción de uso de ciertos antimicrobianos con requisito de autorización por un especialista (ej. infectología) es una estrategia que ayuda a un mejor uso de antibióticos. | totalmente de acuerdo  de acuerdo  neutral  desacuerdo  totalmente en desacuerdo  no aplica |
| 13 | Los antibióticos se usan en exceso (ej. los antibióticos se usan cuando no estan clínicamente indicados) en mi centro de salud. | totalmente de acuerdo  de acuerdo  neutral  desacuerdo  totalmente en desacuerdo  no aplica |
| 14 | La resistencia a los antibióticos es un problema en mi centro de salud. | totalmente de acuerdo  de acuerdo  neutral  desacuerdo  totalmente en desacuerdo  no aplica |
| 15 | En mi establecimiento de salud, se trabaja en equipo para tomar decisiones sobre los antibióticos (ej, dosis, que antibiótico, duración). | totalmente de acuerdo  de acuerdo  neutral  desacuerdo  totalmente en desacuerdo  no aplica |
| 16 | En mi establecimiento de salud, valoro las recomendaciones del PROA para mejorar el uso de antibióticos. | totalmente de acuerdo  de acuerdo  neutral  desacuerdo  totalmente en desacuerdo  no aplica |
| 17 | Tengo acceso a las guías de tratamiento aprobadas localmente en mi establecimiento de salud. | totalmente de acuerdo  de acuerdo  neutral  desacuerdo  totalmente en desacuerdo  no aplica |
| 18 | Existe disponibilidad de consulta a infectología en mi establecimiento de salud. | totalmente de acuerdo  de acuerdo  neutral  desacuerdo  totalmente en desacuerdo  no aplica |
| 19 | Me siento cómodo recomendando cambios a los esquemas de antibióticos iniciados por mis colegas. | totalmente de acuerdo  de acuerdo  neutral  desacuerdo  totalmente en desacuerdo  no aplica |
| 20 | El equipo de salud (ej. médicos, enfermeros, farmacéuticos) educa a los pacientes y/o sus familiares sobre los antibióticos al dar de alta a los pacientes en mi establecimiento de salud. | totalmente de acuerdo  de acuerdo  neutral  desacuerdo  totalmente en desacuerdo  no aplica |
| **PRESCRIBE ANTIBIOTICOS (SI MARCA “SI” CONTINUA, SI MARCA “NO” LA ENCUESTA TERMINA.** | | |
| 21 | Más capacitación sobre los esquemas empíricos, duración de tratamiento y dosis de antibióticos podría mejorar como uso los antimicrobianos en mi establecimiento de salud. | totalmente de acuerdo  de acuerdo  neutral  desacuerdo  totalmente en desacuerdo  no aplica |
| 22 | En mi establecimiento de salud, nos enseñan como seleccionar el mejor esquema de antibióticos basados en los resultados de los cultivos. | totalmente de acuerdo  de acuerdo  neutral  desacuerdo  totalmente en desacuerdo  no aplica |
| 23 | Programas para la optimización del uso de antibióticos (PROA) puede influenciar mis decisiones sobre el inicio y la continuación de los antibióticos en mi establecimiento de salud. | totalmente de acuerdo  de acuerdo  neutral  desacuerdo  totalmente en desacuerdo  no aplica |
| 24 | En mi establecimiento de salud, los pacientes y/o sus familiares demandan antibióticos. | totalmente de acuerdo  de acuerdo  neutral  desacuerdo  totalmente en desacuerdo  no aplica |
| 25 | En mi establecimiento de salud, existe "presión por parte de mis colegas" para prescribir antibióticos. | totalmente de acuerdo  de acuerdo  neutral  desacuerdo  totalmente en desacuerdo  no aplica |
| 26 | La literatura científica influye mis decisiones de como prescribir antibióticos en mi establecimiento de salud. | totalmente de acuerdo  de acuerdo  neutral  desacuerdo  totalmente en desacuerdo  no aplica |
| 27 | La industria farmacéutica influye algunas de mis decisiones sobre la prescripción de antibióticos en mi establecimiento de salud. | totalmente de acuerdo  de acuerdo  neutral  desacuerdo  totalmente en desacuerdo  no aplica |
| 28 | Uso las guías de tratamiento local para decidir que esquema de antibióticos necesitan mis pacientes en mi establecimiento de salud. | totalmente de acuerdo  de acuerdo  neutral  desacuerdo  totalmente en desacuerdo  no aplica |
| 29 | Consulto a infectología o al PROA para decidir el mejor tratamiento empírico para mis pacientes en mi establecimiento de salud. | totalmente de acuerdo  de acuerdo  neutral  desacuerdo  totalmente en desacuerdo  no aplica |
| 30 | Habitualmente obtengo cultivos (cuando es apropiado) antes de comenzar la terapia con antibióticos en mis pacientes. | totalmente de acuerdo  de acuerdo  neutral  desacuerdo  totalmente en desacuerdo  no aplica |
| 31 | Modifico el esquema de antibiótico de mi paciente una vez que recibo los resultados de los cultivos y la susceptibilidad/sensibilidad a los antibióticos. | totalmente de acuerdo  de acuerdo  neutral  desacuerdo  totalmente en desacuerdo  no aplica |
| 32 | Considero los posibles eventos adversos al seleccionar un régimen de antibióticos para mis pacientes. | totalmente de acuerdo  de acuerdo  neutral  desacuerdo  totalmente en desacuerdo  no aplica |
| 33 | Considero las interacciones entre medicamentos cuando selecciono un régimen de antibióticos para una población de pacientes definida en mi establecimiento de salud. | totalmente de acuerdo  de acuerdo  neutral  desacuerdo  totalmente en desacuerdo  no aplica |
| 34 | Considero la función renal de mi paciente al dosificar antibióticos. | totalmente de acuerdo  de acuerdo  neutral  desacuerdo  totalmente en desacuerdo  no aplica |
| 35 | Considero el riesgo de desarrollo de resistencia a los antibióticos en mis pacientes cuando prescribo antibióticos. | totalmente de acuerdo  de acuerdo  neutral  desacuerdo  totalmente en desacuerdo  no aplica |
| 36 | Considero la opinión del personal no médico (por ejemplo, enfermería, farmacia) en la toma de decisiones sobre el uso de antibióticos en mi establecimiento de salud. | totalmente de acuerdo  de acuerdo  neutral  desacuerdo  totalmente en desacuerdo  no aplica |
| 37 | Ser informado sobre la apropiabilidad de los antibióticos que prescribo podría mejorar mi uso de antibióticos. | totalmente de acuerdo  de acuerdo  neutral  desacuerdo  totalmente en desacuerdo  no aplica |
| 38 | Ser informado como mi uso de antibióticos se compara con la de mis compañeros podría mejorar mi uso de antibióticos. | totalmente de acuerdo  de acuerdo  neutral  desacuerdo  totalmente en desacuerdo  no aplica |
| 39 | Soy consciente de los cambios que necesito hacer para mejorar mi uso de antibióticos basado en información que he recibido en mi establecimiento de salud. | totalmente de acuerdo  de acuerdo  neutral  desacuerdo  totalmente en desacuerdo  no aplica |

Participating Hospitals: Instituto de Diagnostico, La Plata, Argentina; Hospital Zonal General de Agudos Dr. Alberto Eurnekian, Buenos Aires, Argentina; Sanatorio Allende Nueva Córdoba, Córdoba, Argentina; Hospital Italiano de Buenos Aires, Buenos Aires, Argentina; Hospital Nacional Profesor Alejandro Posadas, El Palomar, Argentina; Clínica Conciencia, Neuquén, Argentina; Hospital Interzonal General de Agudos San Roque, Buenos Aires, Argentina: Instituto de Cardiología de Corrientes “Juana Francisca Cabral”, Corrientes, Argentina; Hospital Medico Policial Churruca Visca, Buenos Aires, Argentina; Hospital Dr. Marcial V. Quiroga, San Juan, Argentina; Hospital Municipal de Trauma Dr. Federico Abete, Malvinas Argentinas, Argentina; Hospital Angel C. Padilla, Tucumán, Argentina; Hospital El Cruce, Buenos Aires, Argentina; Hospital Alemán, Buenos Aires, Argentina; Hospital Provincial de Rosario, Rosario, Argentina; Hospital Dr. Guillermo Rawson, San Juan, Argentina; Clínica Universitaria Privada Reina Fabiola, Córdoba, Argentina; Hospital Privado Universitario de Córdoba, Córdoba, Argentina; Clinica Privada Provincial, Buenos Aires, Argentina; Maternidad Nuestra Señora De Las Mercedes De Tucumán, Tucumán, Argentina; Hospital San Bernardo, Salta, Argentina; Hospital Municipal de Agudos Dr. Leonidas Lucero, Bahía Blanca, Argentina; Hospital Cesar Milstein, Buenos Aires, Argentina; Clínica De La Mujer, Bogotá, Colombia; Hospital Militar Central, Bogotá, Colombia; Hospital Del Tunal, Bogotá, Colombia; Unidad de Cirugía Cardiovascular de Guatemala, Guatemala City, Guatemala; Hospital San Benito, Peten, Guatemala; Hospital Roosevelt, Guatemala, Guatemala; Hospital Alcivar, Guayaquil, Ecuador; Hospital Metropolitano, Quito, Ecuador; Hospital Sociedad de Lucha Contra el Cáncer, Guayaquil, Ecuador; Hospital Vozandes, Quito, Ecuador; Hospital Carlos Andrade Marín, Quito, Ecuador; Hospital Santo Tomas, Panama, Panama; The Panama Clinic, Panama; Panama; Hospital Punta Pacífica Salud, Panamá, Panama; Hospital Irma de Lourdes Tzanetatos, Panama, Panama; Clinica Hospital San Fernando, Panama, Panama.
